# Fucoxanthinol from the Diatom *Nitzschia Laevis* Ameliorates Neuroinflammatory Responses in Lipopolysaccharide-Stimulated BV-2 Microglia

**DOI:** 10.3390/md18020116

**Published:** 2020-02-17

**Authors:** Yuelian Li, Lu Liu, Peipei Sun, Yifeng Zhang, Tao Wu, Han Sun, Ka-Wing Cheng, Feng Chen

**Affiliations:** 1 Institute for Food and Bioresource Engineering, College of Engineering, Peking University, Beijing 100871, China; 1501111655@pku.edu.cn (Y.L.);; 2 Institute for Advanced Study, Shenzhen University, Shenzhen 518060, China

**Keywords:** *Nitzschia laevis*, fucoxanthinol, anti-inflammation, MAPKs, Akt, NF-κB, Nrf2

## Abstract

In recent years, microalgae have drawn increasing attention as a valuable source of functional food ingredients. Intriguingly, *Nitzschia laevis* is rich in fucoxanthinol that is seldom found in natural sources. Fucoxanthinol, a marine xanthophyll carotenoid, possesses various beneficial bioactivities. Nevertheless, it’s not clear whether fucoxanthinol could exert anti-neuroinflammatory function. In light of these premises, the aim of the present study was to investigate the anti-inflammatory role of fucoxanthinol purified from *Nitzschia laevis* in Lipopolysaccharide (LPS)-stimulated microglia. The results showed that pre-treatment of fucoxanthinol remarkably attenuated the expression of LPS-induced nitric oxide synthase (iNOS) and cyclooxygenase-2 (COX-2), and the production of tumor necrosis factor-alpha (TNF-α), interleukin-6 (IL-6), prostaglandin E2 (PGE-2), nitric oxide (NO) and reactive oxygen species (ROS) induction. Modulation mechanism studies revealed that fucoxanthinol hampered nuclear factor-kappa B (NF-κB), Akt, and mitogen-activated protein kinase (MAPK) pathways. Meanwhile, fucoxanthinol led to the enhancement of nuclear translocation of NF-E2-related factor 2 (Nrf2), and the upregulation of heme oxygenase-1 (HO-1) and NAD(P)H: quinone oxidoreductase 1 (NQO-1). Taken together, the results indicated that fucoxanthinol obtained from *Nitzschia laevis* had great potential as a neuroprotective agent in neuroinflammation and neurodegenerative disorders.

## 1. Introduction

Microglia, the principal primary resident macrophages in the central nervous system (CNS), occupy a pivotal role in the neuroinflammatory responses as well as homeostatic maintenance [1]. In the normal state, microglia serve as phagocytes and function in host protection of the brain [2]. However, aberrantly activated microglia can bring about the release of various pro-inflammatory cytokines and mediators, including nitric oxide (NO), prostaglandin E2 (PGE-2), reactive oxygen species (ROS), interleukin (IL-1β), IL-6, and tumor necrosis factor-alpha (TNF-α), which are closely related to neurodegenerative diseases such as Alzheimer’s disease (AD), Parkinson’s disease (PD), multiple sclerosis, Huntington’s disease, and stroke [3,4,5,6]. Thus, impeding the aberrant activation of microglia is considered an effective therapeutic option for the treatment of neuro-inflammation-related diseases.

Multiple signaling pathways are compactly involved in regulating microglial activation. Among them, nuclear factor-kappa B (NF-κB), a critical transcription factor in modulating microglia-mediated inflammation, consists of P50, P65, and IκB. In response to inflammatory stimuli, IκB phosphorylation and degradation lead to the release of NF-κB dimers into the nucleus, thus expressing specific target genes, such as pro-inflammatory cytokines, inducible nitric oxide synthase (iNOS), and the cyclooxygenase-2 (COX-2) protein [7]. Moreover, the three mitogen-activated protein kinase (MAPK) signaling pathways consisting of c-Jun NH2-terminal kinase (JNK), p38 and extracellular signal-regulated kinase (ERK) also modulate microglial inflammatory responses through the regulation of downstream cellular targets, including NF-κB pathway [8]. In addition, the phosphatidyl inositol 3-kinase/Akt (PI3K/Akt) pathway plays an essential role in the activation of NF-κB and subsequent up-regulation of inflammatory genes expression [8,9]. Therefore, hampering NF-κB, MAPKs, and Akt pathways is widely recognized as a crucial strategy for inhibiting neuroinflammation. 

Reactive oxygen species (ROS), including hydrogen peroxide (H_2_O_2_), superoxide (O_2_^●−^), hydroxide (OH^−^) and hypochlorite (ClO^−^), are closely related to oxidative stress, which is crucial to inflammation [10,11]. In addition, it is known that exaggeratory ROS production is associated with microglial activation and plays an important pathological role in many inflammatory disorders via acting on the NF-κB and MAPKs pathways [12]. Therefore, hampering NF-κB, MAPKs, Akt pathways is widely recognized as a crucial strategy for inhibiting neuroinflammation. In addition, inhibiting ROS production is a potential therapeutic step to suppress the inflammatory response.

It has been well documented that nuclear factor-2 erythroid related factor-2 (Nrf2) is thought to be the prominent signaling modulator in anti-inflammatory defense mechanisms and neuroprotection. Nrf2 modulates the transcription of antioxidant and cytoprotective genes such as heme oxygenase-1 (HO-1) and NAD (P) H: quinone oxidoreductase 1 (NQO-1). HO-1 is essential for decreasing the levels of COX-2 and iNOS, which was recognized as a potential target for intervening neuro-inflammation. NQO-1, clearly induced or repressed phase II enzyme, is implicated in protection against oxidative stress [13,14]. Thus, activating the Nrf2/HO-1/NQO-1 pathway is also suggested as a good target for the treatment of neuroinflammatory diseases. 

**Figure 1:**
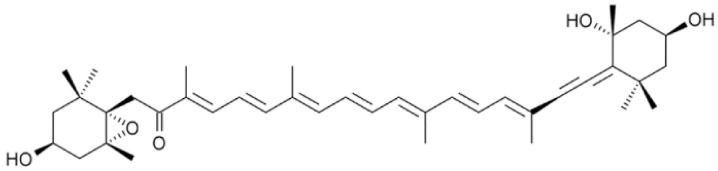
Chemical structure of fucoxanthinol.

Fucoxanthinol (Fuol), a marine xanthophyll carotenoid, possesses unusual structures with an allelic bond, a conjugated carbonyl, and a 5, 6-monoepoxid (Figure 1). Fuol is a major gastrointestinal metabolite of dietary fucoxanthin. To date, there are few reports about natural sources for the preparation of this compound. *Nitzschia laevis*, a diatom, is rich in Fuol and can be cultivated on a large scale through heterotrophic methods with a high growth rate [15,16]. Therefore, *N. laevis* possesses the potential to be a candidate for industrial production of Fuol. 

Recent reports demonstrate that fucoxanthin possesses anti-inflammatory effects with significant attenuation of inflammatory mediators, including NO, iNOS, COX-2, and ROS in RAW264.7 macrophages [17], microglial cells [18], human lymphocytes [19]. Besides, fucoxanthin could also inhibit the inflammation response via the down-regulation of NLR family pyrin domain containing-3 (NLRP3) inflammasome and IL-1β production [20]. Whereas, fucoxanthin does not exhibit the biofunctional activities in the body and is metabolized into the functional form of Fuol in gastrointestinal and intestinal tracts, and then released into the blood circulation [21,22]. Additionally, Fuol has been demonstrated to show the superior functional effects in contrast with fucoxanthin. For instance, Fuol exerted a more potent antiproliferative effect on prostate cancer cells, human T-cell leukemia virus type 1 cells, and 3T3-L1 cells than fucoxanthin [23,24,25]. Moreover, Fuol possesses various beneficial biological activities such as antioxidation [26], prevention of different types of cancers [27], and protection of central nervous system neurons [28]. Although some reports suggest that fucoxanthin could alleviate TBI-induced secondary brain injury and attenuate scopolamine-induced cognitive impairments in mice, seldom reports have claimed the role of Fuol in those brain diseases [29,30]. In particular, the effects of Fuol on neuroinflammation in microglia and neuroinflammatory-related diseases are unclear. Thus, the present study aimed to investigate the anti-inflammatory activity and possible mechanism of Fuol purified from *N. laevis* in Lipopolysaccharide (LPS)-activated BV-2 microglia cells.

## 2. Results

### 2.1. Cytotoxic Effect of Fucoxanthinol on BV-2 Cells 

**Figure 2:**
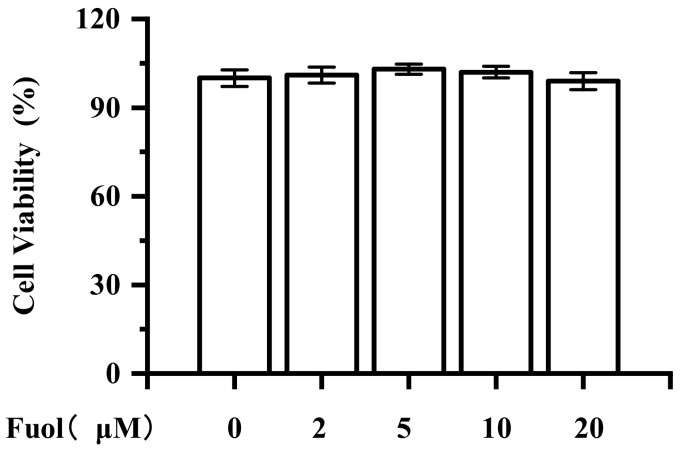
Effects of fucoxanthinol on BV-2 microglial cell viability. Cells were treated with various concentrations (2, 5, 10, and 20 μM) of Fuol for 24 h. Then, the cytotoxicity of Fuol was measured by the cell counting kit-8 (CCK-8) assay and data were normalized as % of control. Values are presented as the means ± SD of five independent experiments in triplicate.

To evaluate the effects of Fuol on BV-2 cell viability, a cell counting kit-8 (CCK-8) assay was carried out with BV-2 cells. As shown in Figure 2, after 24 h of incubation with different concentrations of Fuol (from 2 μM up to 20 μM), the cell viability of BV-2 cells was not appreciably altered by any doses of Foul treatment. LPS (1 μg/mL) decreased the cell viability to 82.61%, while pretreatment with Fuol (20 μM) increased the cell viability to 88.19% (Figure S1). Thus, concentrations of Fuol from 5 to 20 μM were used in the subsequent experiments.

### 2.2. Fucoxanthinol Inhibited LPS-Induced iNOS, NO, PGE-2, and COX-2 Expression in BV-2 Cells 

**Figure 3:**
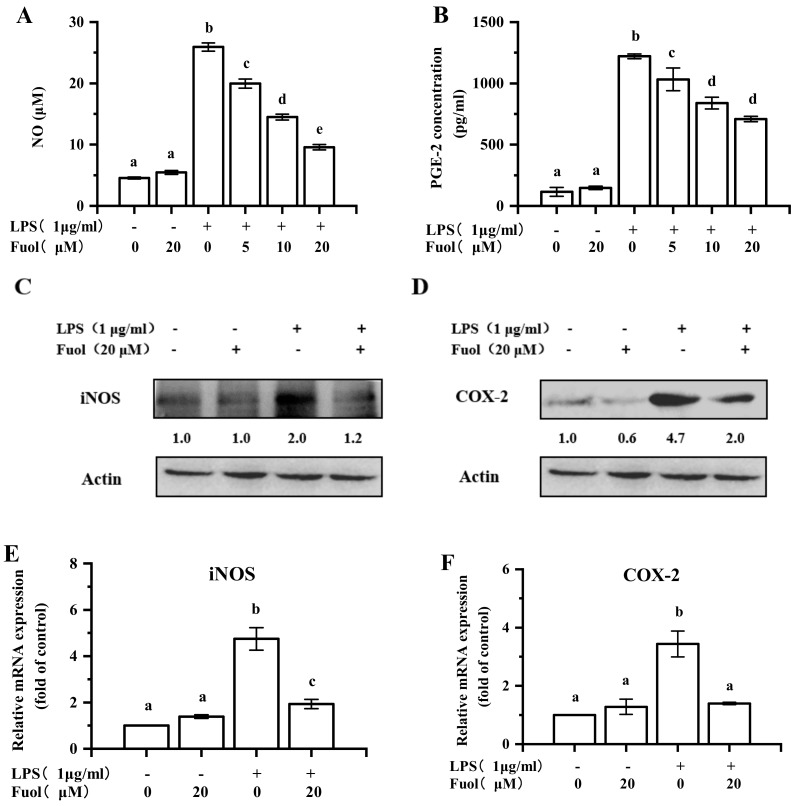
Fucoxanthinol inhibited LPS-induced production nitric oxide (NO) and prostaglandin E2 (PGE-2), and the expression of inducible nitric oxide synthase (iNOS) and cyclooxygenase-2 (COX-2) in BV-2 cells. BV-2 cells were pretreated with Fuol (5, 10, and 20 μM) for 4 h and then incubated with Lipopolysaccharide (LPS) (1 μg/mL) for 24 h. (**A**) The NO concentration in the supernatant was determined by using Griess reagent. (**B**) The secretory levels of PGE-2 in supernatants were measured by using an ELISA kit. (**C**,**D**) BV-2 cells were pretreated with Fuol (20 μM) for 4 h and then incubated with LPS (1 μg/mL) for 24 h, iNOS, and COX-2 protein expressions were measured by Western blotting. (**E**,**F**) BV-2 cells were pretreated with Fuol (20 μM) for 4 h and then incubated with LPS (1 μg/mL) for 6 h. Then, mRNA was extracted, and the mRNA level of iNOS and COX-2 was evaluated by real-time reverse transcription-PCR (RT-PCR). The results were presented as means ± SD of three independent experiments. Different letters in the columns indicate a significant difference (*p* < 0.05). Two columns not sharing the same letter are significantly different.

NO, an important mediator in the inflammatory response, was determined by Griess reagent. As shown in Figure 3A, stimulation with LPS led to a marked increase in NO production. In contrast, Fuol significantly suppressed the production of NO in a dose-dependent manner. It is generally known that NO production is mainly dependent on the iNOS expression. Accordingly, we explored the effects of iNOS expression. As shown in Figure 3C,E, the expression of iNOS was notably inhibited by Fuol at both protein level and mRNA level. PGE2, a mediator of inflammation as well, is produced from LPS-induced endogenous arachidonic acid by COX-2 in stimulated microglial cells. As illustrated in Figure 3B, Fuol treatment resulted in evident attenuation of the secretory levels of PGE-2 in a dose-dependent manner. Meanwhile, Fuol markedly hampered COX-2 protein expression (Figure 3D) and mRNA transcription (Figure 3F).

### 2.3. Fucoxanthinol Ameliorated LPS-induced Pro-Inflammatory Cytokines Production in BV-2 Cells

**Figure 4:**
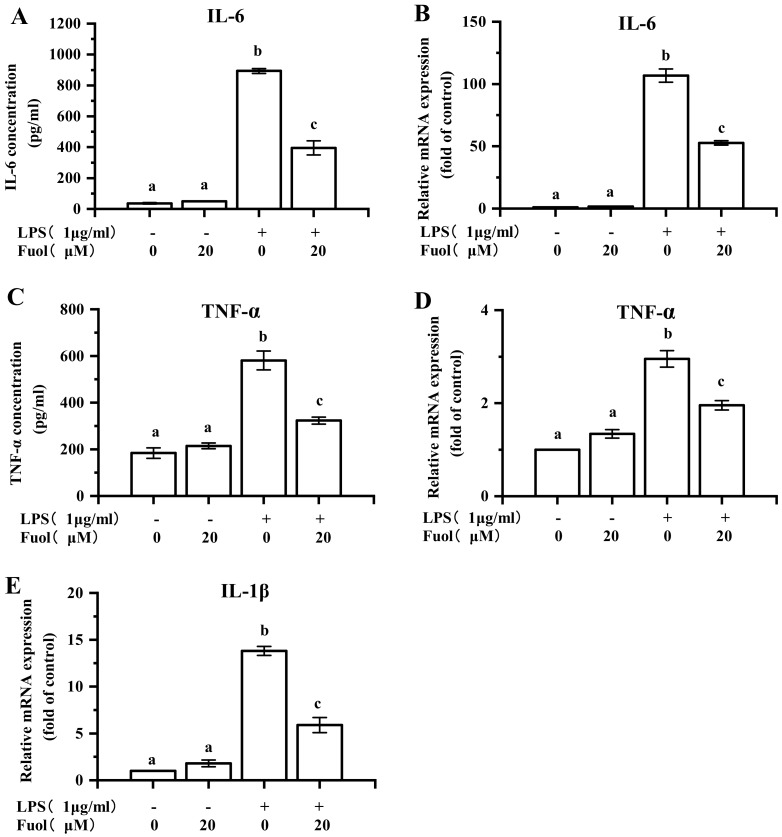
Fucoxanthinol decreased LPS-induced pro-inflammatory cytokines production. (**A**,**C**) BV-2 cells were pretreated with Fuol (20 μM) for 4 h and then treated with LPS (1 μg/mL) for 24 h. The secretory levels of TNF-α and IL-6 in supernatants were measured using ELISA. (**B**,**D**,**E**) BV-2 cells were incubated with Fuol (20 μM) for 4 h and followed by LPS treatment for 6 h. Then, mRNA was extracted and the mRNA level of IL-6 (**B**), TNF-α (**D**), and IL-1β (**E**) was evaluated by RT-PCR. The results were exported as a fold change against controls and was presented as means ± SD of three independent experiments. Different letters in the columns indicate a significant difference (*p* < 0.05). Two columns not sharing the same letter are significantly different.

It has been recognized that LPS stimulation in BV-2 cells evokes various pro-inflammatory cytokines secretion, including IL-1β, IL-6, TNF-α, and etc. Herein, we assessed the effects of Fuol on the LPS induced pro-inflammatory cytokines production. As exhibited in Figure 4A,C, compared with control, LPS stimulation induced an evident increase of IL-6, TNF-α in the cell culture supernatant via ELISA method determination. However, the pretreatment with Fuol impaired the production of pro-inflammatory cytokines when compared to the LPS challenge. Ulteriorly, we determined whether Fuol could hinder the mRNA expression of IL-6 and TNF-α. Consistently, the pretreatment of Fuol effectively impeded the mRNA expression of IL-6 and TNF-α induced by LPS in BV-2 cells (Figure 4B,D). In addition, we examined the mRNA expression of IL-1β, Fuol significantly impaired the up-regulation of IL-1β by LPS treatment accordingly (Figure 4E). These data showed that Fuol suppressed the LPS-induced production of inflammatory cytokines in BV-2 cells.

### 2.4. Fucoxanthinol Repressed the Nuclear Translocation of NF-κB

**Figure 5:**
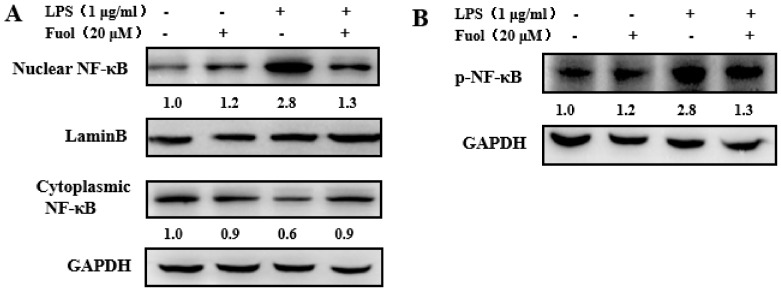
Fucoxanthinol suppressed LPS-induced nuclear factor-kappa B (NF-κB) activation. (**A**,**B**) BV-2 cells were preincubated with Fuol (20 μM) for 4 h and then added LPS (1 μg/mL) for 1 h. (**A**) The protein expression of NF-κB p65 in the nucleus and cytoplasm was analyzed by the Western blot method. (**B**) Total cell extracts were subjected to Western blot analysis using an antibody against phospho-NF-κB P65.

To further investigate the mechanism underlying the anti-inflammatory effects of Fuol, we concentrated on the NF-κB signaling pathway. NF-κB, a primary regulator of genes, plays an important role in the induction of pro-inflammatory cytokines. Thus, we investigated whether Fuol hindered the NF-κB signaling pathway. For this purpose, we first analyzed the effect of Fuol on NF-κB p65 phosphorylation level in whole-cell extracts. As shown in Figure 5B, the stimulation of BV-2 cells with LPS led to the marked increase of NF-κB p65 level phosphorylation, which could be notably reduced by Fuol pretreatment. Then, we examined the expression of NF-κB p65 in the nucleus and cytoplasm. As shown in Figure 5A, LPS stimulation resulted in decreased NF-κB p65 level in the cytoplasm and increased NF-κB p65 level in nucleus compared with unstimulated cells. In contrast, pretreatment with Fuol attenuated the NF-κB p65 level in the nucleus, which indicated that Fuol treatment inhibited NF-κB p65 translocation from the cytoplasm into the nucleus. Collectively, our results demonstrate that Fuol inhibited LPS stimulated inflammation response via the NF-κB pathway. 

### 2.5. Fucoxanthinol Inhibited LPS-Stimulated MAPKs and PI3K/Akt Pathways Activities 

**Figure 6:**
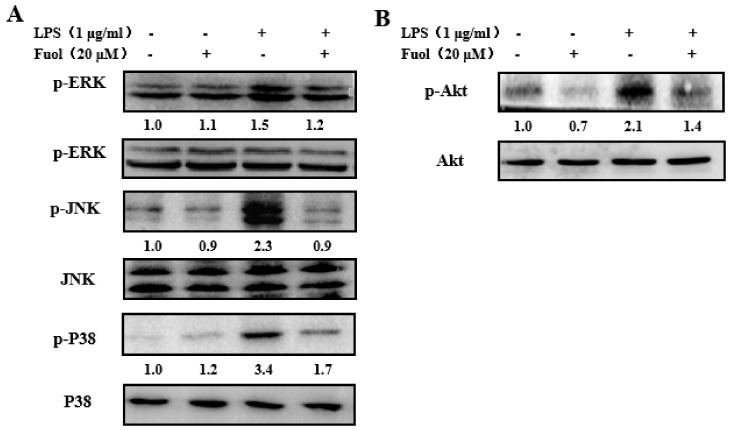
Fucoxanthinol inhibited LPS-induced phosphorylation of MAPKs and Akt. (**A**,**B**) BV-2 cells were pretreated with Fuol (20 μM) for 4 h then exposed to LPS (1 μg/mL) addition for 1 h. Then, total cell extracts were subjected to Western blot analysis using antibodies against phospho- or total forms of extracellular signal-regulated kinase (ERK), c-Jun NH2-terminal kinase (JNK), p38, and Akt.

Previous studies have demonstrated MAPKs involved in the regulation of the NF-κB pathway in microglia [8]. Therefore, we examined the effect of Fuol on MAPKs activation. As exhibited in Figure 6A, LPS treatment markedly promoted the phosphorylation of MAPKs, including ERK, JNK, and P38 compared to the control, along with the unaltered basal expression level of corresponding MAPKs. Whereas Fuol effectively ameliorated phosphorylation of MAPKs, which indicated Fuol blocked the MAPKs pathway and then inhibited the LPS-induced inflammatory responses in BV-2 cells. Furthermore, the PI3K/Akt signal pathway plays a necessary role in the NF-κB-mediated inflammatory response as well, thus, we investigated the influence of Fuol on Akt phosphorylation. As shown in Figure 6B, LPS stimulation increased Akt phosphorylation in comparison with the untreated group, without changing the basal level of Akt. Inversely, Fuol significantly reversed the Akt phosphorylation level. Our results suggested that Fuol impeded the LPS-stimulated inflammatory response via the Akt signaling pathway.

### 2.6. Effects of Fucoxanthinol on the Accumulation of ROS, HO-1 and NQO-1 and the Activation of Nrf2 

**Figure 7:**
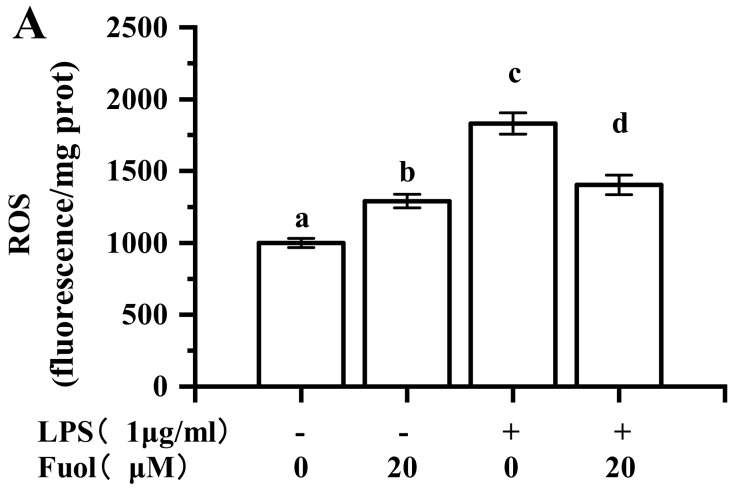

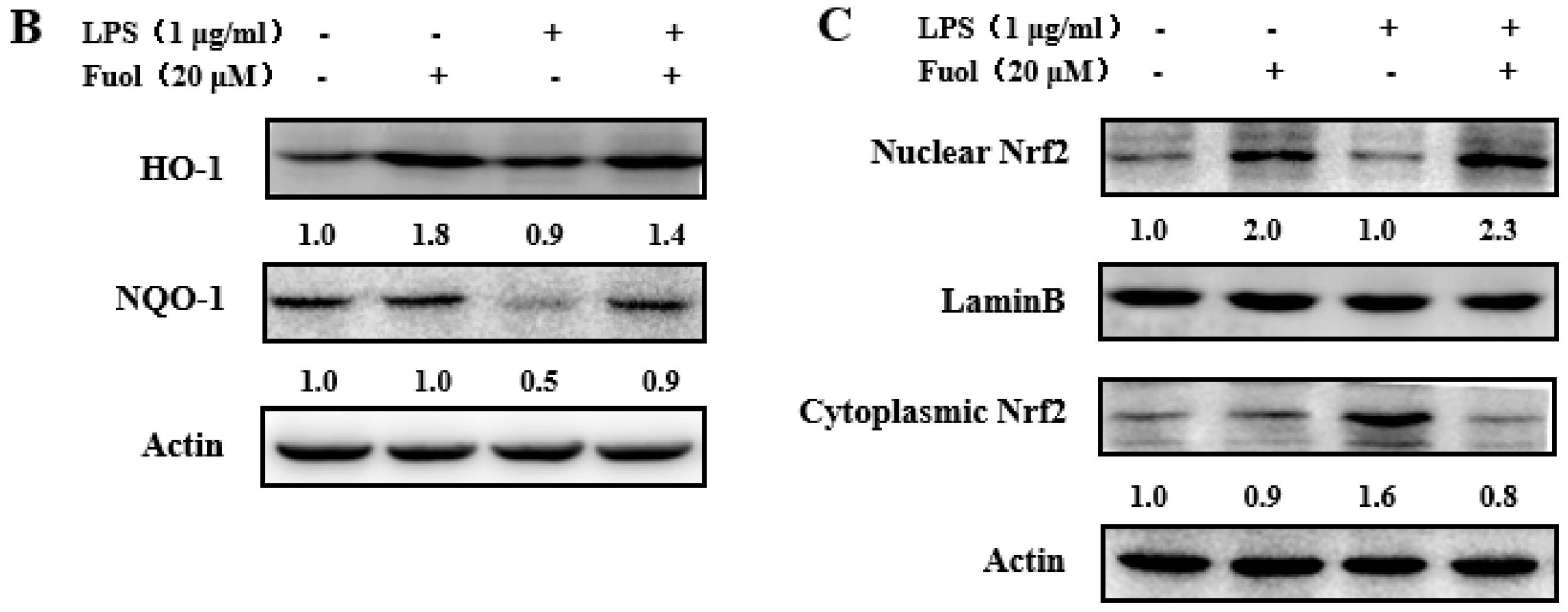
Effects of fucoxanthinol on the production of reactive oxygen species (ROS), heme oxygenase-1(HO-1), and NAD(P)H: quinone oxidoreductase 1(NQO-1), and the activation of Nrf2. (**A**) BV-2 cells were pretreated with Fuol for 4 h and then exposed to LPS (1 μg/mL) for 6 h. Then ROS levels were determined by DCFH oxidation according to the manufacture’s instructions. (**B**) BV-2 cells were pretreated with Fuol for 4 h and then exposed to LPS for 12 h. Then, total cell lysates were subjected to Western blot analysis using antibodies against HO-1 and NQO-1. (**C**) Nuclear and cytosolic extracts were subjected to Western blot analysis using an antibody against Nrf2. Different letters in the columns indicate a significant difference (*p* < 0.05). Two columns not sharing the same letter are significantly different.

ROS, are involved in microglia activation. This finding demonstrated that Fuol could attenuate the LPS-induced increase of the intracellular ROS in BV-2 cells (Figure 7A). Nrf2 has been recognized as the prominent signaling mediator modulating redox homeostasis and executing anti-inflammatory effects through regulating cytoprotective enzymes and the related proteins, including HO-1 and NQO-1. As shown in Figure 7B, Fuol alone pretreatment group markedly up-regulated the protein expression of HO-1 and NQO-1 in comparison with the control group. Besides, the LPS-stimulated group resulted in a decrease in HO-1 and NQO-1 expression, which remarkably increased after Fuol pretreatment. We further examined the effect of Fuol on Nrf2 translocation in BV-2 cells. Fuol promoted the translocation of Nrf2 into the nucleus and a decrease in the cytoplasm compared to untreated cells. In addition, Fuol pretreatment ameliorated the decline in LPS induced nuclear accumulation of Nrf2 and reduced the Nrf2 expression in the cytoplasm compared with LPS treatment (Figure 7C). In general, our results suggest that Fuol attenuates neuro-inflammation partly via promoting the Nrf2 antioxidant protective mechanism in BV-2 microglia. 

## 3. Discussion

*N. laevis* has been reported to possess the potential as a biological resource. Alzahrani exhibited the antioxidant, anti-ACE, and anti-AChE activities of aqueous protein extracts from *N. laevis*, indicating the possible new source with nutraceutical and pharmaceutical potentials [31]. Besides, unsaturated fatty acids and carotenoids such as fucoxanthin and Fuol from *N. laevis* contributed to the strong inhibitory capacities against the formation of total advanced glycation endproducts (AGEs) [32,33]. Meanwhile, Fuol has been demonstrated to play an important role in anti-inflammatory effects. For instance, Fuol could inhibit the production of inflammatory cytokines such as nitric oxide and PGE2 in abdominal white adipose tissue [34]. Although Fuol has been reported to possess the ability to protect central nervous system neurons, the potential mechanisms of Fuol in the context of the neuroprotective effects are not yet well understood. Microglia are poised to provide first line of defense in the CNS to initiate the immune response. Aberrant and prolonged activation of microglia is engaged in multiple neurodegenerative disorders [35]. In consequence, there is a strong need to investigate whether Fuol exhibited the anti-inflammatory function in microglia. Our founding suggested that Fuol acquired from *N. laevis* warranted remarkable anti-neuroinflammatory properties since it inhibited the production of multiple pro-inflammatory mediators, including NO, PGE-2, ROS, IL-1β, TNF-α, IL-6, COX-2, iNOS in LPS-induced BV-2 cells (Figure 3, Figure 4 and Figure 7A). 

We further investigated the molecular mechanism of Fuol on modulating inflammatory response in LPS-stimulated BV-2 cells. NF-κB, an important transcription factor, plays a vital role in neuroinflammation. NF-κB is able to modulate the transcription of a range of host genes involved in inflammatory and immune responses during neurodegeneration [36,37]. As suggested in our study, Fuol significantly inhibited the activation of the NF-κB pathway via suppressing the phosphorylation and nuclear translocation of p65 NF-κB in LPS-stimulated BV-2 cells (Figure 5). Moreover, PI3K/Akt and MAPKs signaling are known as critical regulators for NF-κB-mediated inflammatory responses [38,39]. MAPKs form three-tiered kinase cascades resulting in the activation of the effector kinases ERK, JNK, and p38 [40]. It has been demonstrated that JNK activation is strongly implicated in inflammatory responses, neurodegeneration, and apoptosis. Moreover, JNK occupies a crucial role in the induction of apoptosis in neurons. Thus, the previous report indicated that modulation of JNK pathway could protect against cerebral ischemia and other neurodegenerative conditions [41,42]. The p38 MAPK is a stress-activated kinase and is regarded as an important target of pro-inflammatory cytokines and oxidative stress in microglia [43]. The ERK1/2 pathway is known to participate in cell proliferation, differentiation, and cell survival. In addition, the ERK1/2 pathway could be triggered by phosphorylation in response to various cytotoxic stress stimuli in the brain [44,45]. In this work, Fuol pre-treatment markedly prevented the LPS-induced activation of JNK, p38, and ERK1/2 (Figure 6). Thus, the intervention of MAPK pathways might partially elucidate anti-inflammatory mechanisms of Fuol. Akt, a serine/threonine kinase, is activated by PI3K. It has been reported that PI3K/Akt pathway is engaged in various cellular processes such as apoptosis, inflammatory responses, differentiation, and tumor angiogenesis [46,47,48]. As the upstream gene of NF-κB, Akt could modulate IκB degradation via phosphorylation of IKK in activated BV-2 microglia [49]. We demonstrated that Fuol could intervene in inflammatory responses by inhibiting the phosphorylation of Akt signal pathways in LPS-induced BV-2 cells. Therefore, the NF-κB, MAPKs, and PI3K/Akt are potential targets for the prevention of inflammatory response. 

At low to moderate concentrations, ROS serve as an important second messenger inside the cell and commonly are produced during inflammatory responses. In inflammatory responses, LPS induces the production of ROS via NADPH oxidase activation, and results in the activation of the MAPK signaling pathway. Moreover, excess ROS could overwhelm normal antioxidant capacity, which results in perturbation of cellular redox balance [50]. Nrf2 is the primary transcription factor required for cellular defense against oxidative stress and functions as a vital regulator in cellular defense against endogenous or exogenous stress caused by ROS [51]. Once activated, Nrf2 translocates from the cytoplasm to the nucleus and then binds to the antioxidant response element, thus activating the transcription of various downstream genes, including HO-1, NQO-1, and γ-GCLC [14]. Furthermore, accumulating evidence has suggested that Nrf2 and the subsequent transcription of Nrf2 related antioxidant proteins are complicated in attenuating inflammation-associated pathogenesis by inhibiting the production of pro-inflammatory cytokines and the expression of iNOS and COX-2. Moreover, some reports demonstrate that HO-1 inhibits LPS-stimulated inflammatory responses by blocking NF-κB activity [52,53,54]. In the present study, Fuol significantly inhibited the production of ROS and enhanced the nuclear translocation of Nrf2 to induce HO-1 and NQO-1 expression (Figure 7). Hence, Nrf2/ HO-1/ NQO-1 pathway is a vital target for an anti-inflammatory response. 

There might be a coordinated crosstalk mechanism between two crucial transcription factors, NF-κB and Nrf2. Nrf2 activation could induce intracellular events that result in NF-κB suppression. On the other hand, the repressed activity of Nrf2 could promote NF-κB action, leading to the increase of inflammatory cytokine production. The transcription factors exhibit adverse effects on the target gene expression [55,56]. For example, Nrf2 deficient mice have aggravated the activation of NF-κB in response to LPS stimulation [56]. In the present study, our results also showed the inverse relationship between the two transcription factors. Further studies will be necessary to understand the definitive mechanisms of Fuol in modulating Nrf2 and NF-κB. Besides, recent evidence proves that Nrf2 modulates anti-inflammatory responses through the MAPKs pathway [57]. Some reports also demonstrate that ERK1/2 is an important signaling protein implicated in Nrf2 activation [58]. In the present study, Fuol significantly suppressed LPS-stimulated the activation of NF-κB and MAPK and promoted the activation of Nrf2. Although Fuol could modulate multiple pathways, however, the direct targets and the definitive mechanisms of Fuol are currently unclear and need to be further explored in the future.

In addition, recent accumulating evidence have revealed that Fuol is a safe compound and induces no side effects. Some reports suggest that fucoxanthin-rich functional foods are used for anti-obesity treatments in Western countries [21]. Thus, the dietary of Fuol isolated from *N. laevis* might have the potential as a promising marine drug. More well-controlled clinical trials deserve further investigation. Taken together, we suggest that Nrf2, NF-κB, MAPKs, and Akt signaling are involved in the anti-inflammatory of Fuol in LPS-induced microglia. These evidence indicate that Fuol obtained from *N. laevis* possesses the potential as a nutritional preventive strategy in inflammation-related neurodegenerative diseases. 

## 4. Materials and Methods 

### 4.1. Chemicals and Antibodies

Lipopolysaccharide (LPS) was purchased from Sigma (St. Louis, MO, USA) and dissolved in PBS for use. Nitric oxide (NO) assay kit was purchased from Beyotime Institute of Biotechnology (Shanghai, China). Enzyme-linked immunosorbent assay (ELISA) kits for TNF-α, PGE-2, and IL-6 were obtained from MultiSciences Company (Hangzhou, China).

Antibodies against COX-2 (4842), iNOS (2982), β-actin (3700), NF-κB p65 (8242), p-p44/42 MAPK (ERK1/2) (9101), p44/42 MAPK (ERK1/2) (9102), pSAPK/JNK (Thr183/Tyr185) (9251), SAPK/JNK (9252), HO-1 (43966) and NQO-1 (3187) were purchased from Cell Signaling Technology Company (Shanghai, China). All of the chemicals were of analytical grade.

### 4.2. Isolation of Fucoxanthinol from Diatom Nitzschia Laevis

The diatom *N. laevis* (UTEX 2047) was obtained from the University of Texas Culture Collection (Texas, USA). Cells were cultured in modified Lewin’s marine diatom medium. After cultivation, freeze-dried powders were extracted by methanol to obtain pigments, and then Fuol was isolated by solid-phase extraction (SPE), as the previous study described [15].

### 4.3. Cell Culture and Treatment

Mouse microglial (BV-2) cells were obtained from the China Center for Type Culture Collection (Beijing, China) and incubated in DMEM medium with 10% fetal bovine serum and 100 units/mL penicillin-streptomycin at 37 °C in a humidified incubator (5% CO_2_, 95% air). The BV-2 cells were selected from the third to the fifteenth generation for experiments. Fuol were dissolved in dimethyl sulfoxide, LPS (1 μg/mL) was dissolved in phosphate buffer (0.01 M, pH 7.2).

### 4.4. Cell Viability Assays

Cell viability was determined by the CCK-8 cell counting kit (Vazyme). BV-2 cells were plated into 96-well culture plates (1.0 × 10^5^ cells per well) in 100 μL volume and incubated at 37 °C for 24 h. The culture medium was subsequently replaced by medium containing different concentrations of Fuol (0, 2, 5, 10 and 20 μM). At the point of 24 h, cells in each well were incubated with 100 μL of culture medium containing 10 μL CCK-8 reagent at 37 °C for 4 h. Then, the absorbance of each well was determined at 450 nm using a microplate reader.

### 4.5. Nitric Oxide Assays

The production of NO was determined by the Griess reagent as the previous study described [59]. BV-2 cells were cultured in 6-well culture plates and pretreated with the indicated concentrations of Fuol 4 h prior to stimulation with LPS (1 μg/mL) for 24 h. Cell supernatants were collected and assayed for NO production. Nitrite production was measured by adding 100 μL of cell culture medium to 100 μL of Griess reagent [0.1% (*w*/*v*) N-(1-naphthyl)-ethylenediamine and 1% (*w*/*v*) sulfanilamide in 5% (*v*/*v*) phosphoric acid] in a 96-well plate and incubating in the dark for 15 min. The absorbance of each well was determined at 540 nm using a microplate reader.

### 4.6. Real-Time Quantitative Reverse Transcription Polymerase Chain Reaction (RTPCR)

Total RNA was extracted by using the MiniBEST Universal RNA Extraction Kit (TaKaRa Bio, Inc., Kyoto, Japan), according to the manufacturer’s instructions. The concentration and purity of all RNA samples were determined using a Nanodrop spectrophotometer (Nanophotometer NP 80, Implen, Germany). RNA samples were stored at −80 °C.

Total RNA (500 ng) was extracted from different samples and reverse transcribed to synthesize cDNA by using a PrimeScript RT Master Mix Kit (TaKaRa Bio, Inc., Kyoto, Japan). One microliter of diluted cDNA was subjected to real-time PCR by the CFX96 Real-Time PCR Detection System (BioRad, Shanghai, China). PCR was performed in a 25 μL volume using SYBR^®^ Premix Ex Taq™ II (TaKaRa Bio, Inc., Kyoto, Japan) under the following conditions: an initial denaturation at 95 °C for 30 s, 39 cycles (95 °C for 5 s, 55 °C for 30 s, 72 °C for 30 s) of DNA amplification, followed by a final extension (72 °C) for 10 min. All primers were purchased from Sangon Biotech. All PCR assays were performed in triplicate. The reaction mixtures without template cDNA were used as negative controls. The Primers used for real-time PCR are listed in Table 1.

### 4.7. Enzyme-Linked Immunosorbent (ELISA) Assay

BV-2 cells were seeded in 6-well plates (3.75 × 10^6^ cells per well) and pretreated with the indicated concentrations of Fuol prior to stimulation with LPS (1 μg/mL) for 24 h. Then, cell supernatants were collected and centrifuged at 13,000 rpm for 10 min. The levels of TNF-α, PGE-2, and IL-6 were measured by the ELISA kits according to the manufacturer’s instruction (R&D Systems, Minneapolis, MN, USA; eBioscience, San Diego, CA, USA).

### 4.8. Western Blot Analysis

Proteins were extracted from the BV-2 cells. Protein concentrations were determined using a BCA protein assay kit (Beyotime, Shanghai, China). The proteins were subjected to 10% SDS-polyacrylamide gel electrophoresis (SDS/PAGE) and electrotransferred onto nitrocellulose membranes (0.45 μm Millipore) using a Wet/Tank Blotting Systems (Bio-Rad, Shanghai, China). For Western blotting analysis, membranes were incubated with primary antibodies overnight at 4 °C followed by incubation with a secondary antibody for 1 h at 25 °C. Then the blots were developed with the chemiluminescent substrate and then detected by Amersham Imager 600 (G.E, USA). The equivalent loading of proteins in each well was confirmed by GAPDH, β-actin, or LaminB control.

### 4.9. Detection of Intracellular ROS Production

BV-2 cells were cultured in 6-well plates and treated with LPS (1 μg/mL) in the presence or absence of indicated dose of Fuol at 37 °C for 6 h. After incubation, 10 μM 2′, 7′-dichlorofluorescein diacetate (DCFH-DA) was added and incubated for 30 min at 37 °C. Then, cells were washed twice with PBS (0.01 M, pH 7.4) and immediately harvested and lysed with cell lysis buffer (Beyotime Institute of Biotechnology, Jiangsu, China). The fluorescence of the supernatant of the cell extract was read at excitation/emission wavelengths of 485/525 nm by using a fluorescence microplate reader. 

### 4.10. Statistical Analysis

All measurements were performed in triplicate. The data were analyzed by using one-way analysis of variance followed by Tukey’s multiple-range posthoc test with the SPSS Statistics 17.0 software (IBM Corporation, Armonk, NY, USA). The results are presented as mean ± SD from at least three independent experiments. A *p*-value of less than 0.05 was considered to be statistically significant.

## 5. Conclusions

This study is the first to demonstrate the anti-neuroinflammatory effects of Fuol in LPS-induced BV-2 cells. The results showed that Fuol effectively suppressed the production of inflammatory mediators and cytokines in BV-2 cells by blocking NF-κB, Akt, MAPKs pathways, and promoting Nrf2/NQO-1/HO-1 pathways. These results provided insights into the mechanism underlying the anti-neuroinflammatory effects of Fuol and also indicated the potential of Fuol in the treatment of inflammatory and neurodegenerative disorders such as AD which associate with microglia activation and neuroinflammation. The results in the present study provided strong evidence that fucoxanthinol obtained from *N. laevis* could exert the potential protective effect against the neuroinflammatory response in BV-2 cells.

## Figures and Tables

**Table 1:** RT-PCR primers used for real-time PCR.

Genes	Forward (5′–3′)	Reverse (5′–3′)
iNOS	GAAGAAAACCCCTTGTGCTG	GTCGATGTCACATGCAGCTT
COX-2	GATGTTTGCATTCTTTGCCC	TGAAGCCATGACCTTTCGCATTAGCATGG
TNF-α	GAAAAGCAAGCAGCCAACCA	CGGATCATGCTTTCTGTGCTC
IL-1β	AATGACCTGTTCTTTGAAGTTGA	TGATGTGCTGCTGCGAGATTTGAAG
IL-6	ACAAGTCGGAGGCTTAATTACACAT	TTGCCATTGCACAACTCTTTTC
β-actin	TCCTCCTGAGCGCAAGTACTCT	GCTCAGTAACAGTCCGCCTAGAA

## Supplementary Materials

The following are available online at https://www.mdpi.com/1660-3397/18/2/116/s1, Figure S1: Effect of fucoxanthinol on cell viability in LPS-activated BV-2 cells.
