# Supplementary material for: Fucoxanthinol from the Diatom Nitzschia Laevis Ameliorates Neuroinflammatory Responses in Lipopolysaccharide-Stimulated BV-2 Microglia
Source: Mar Drugs. 2020 Feb 17;18(2):116. doi: 10.3390/md18020116 (PMC7074591; doi:10.3390/md18020116)
Supplement: Supplementary file 1 [file marinedrugs-18-00116-s001.pdf]

Supplementary Information

Fucoxanthinol from the diatom *Nitzschia laevis* ameliorates neuroinflammatory responses in lipopolysaccharide-stimulated BV-2 microglia

Yuelian Li[1], Lu Liu[1, 2], Peipei Sun[1], Yifeng Zhang[1], Tao Wu[1], Han Sun[1, 2], Ka-Wing Cheng[2], Feng Chen [2*]

1 Institute for Food and Bioresource Engineering, College of Engineering, Peking University, Beijing 100871, China; 1501111655@pku.edu.cn (Y.L.); ll652866844@163.com (L.L.); zixinsp@163.com (P.S.); yifengzhang@pku.edu.cn (Y.Z.); 1501111658@pku.edu.cn (T.W.); shlyg2242@163.com (H.S.);

2 Institute for Advanced Study, Shenzhen University, Shenzhen 518060, China; shlyg2242@163.com (H.S.); kcheng2018@126.com (K.-W.C); sfchen@szu.edu.cn and sfchencoe@pku.edu.cn (F.C.)

* Correspondence: sfchen@szu.edu.cn and sfchencoe@pku.edu.cn; Tel.: +86 10 62745356 (F.C.); ORCID: 0000-0002-9054-943X

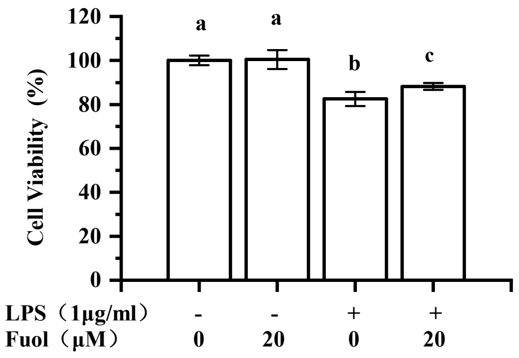

Figure S1 Effect of fucoxanthinol on cell viability in LPS-activated BV-2 cells. BV-2 cells were pretreated with fucoxanthinol (20 μM) for 4 h, and then incubated with LPS (1 μg/mL) for 24 h. Then, the cytotoxicity of Fuol was measured by CCK-8 assay and data were normalized as % of control. Values are presented as the means ± SD of five independent experiments in triplicate. Different letters in the columns indicate significant difference ($p<0.05$).
